# Perception of speech rhythm in second language: the case of rhythmically similar L1 and L2

**DOI:** 10.3389/fpsyg.2015.00316

**Published:** 2015-03-25

**Authors:** Mikhail Ordin, Leona Polyanskaya

**Affiliations:** Fakultät für Linguistik und Literaturwissenschaft, Universität BielefeldBielefeld, Germany

**Keywords:** speech rhythm, rhythm metrics, durational variability, rhythm acquisition, rhythm perception, timing patterns, rhythm development, second language

## Abstract

We investigated the perception of developmental changes in timing patterns that happen in the course of second language (L2) acquisition, provided that the native and the target languages of the learner are rhythmically similar (German and English). It was found that speech rhythm in L2 English produced by German learners becomes increasingly stress-timed as acquisition progresses. This development is captured by the tempo-normalized rhythm measures of durational variability. Advanced learners also deliver speech at a faster rate. However, when native speakers have to classify the timing patterns characteristic of L2 English of German learners at different proficiency levels, they attend to speech rate cues and ignore the differences in speech rhythm.

## Introduction

The differences between languages and linguistic varieties are manifested in the acoustic components of the signal that are perceived by the auditory system and cognitively processed to extract linguistic structures. Some minute acoustic differences are perceived by the native speakers, while some gross acoustic changes in the speech stream may be ignored—either not perceived not attended to. In this study we concentrated on the perceptual relevance of the changes in speech rhythm in second language (L2) that happen in the course of L2 acquisition, provided that the native and the target languages of the learner are rhythmically similar.

We start by introducing the notion of rhythm. Further we move on to discussing why perception of rhythmic patterns might be linguistically relevant. Then we report how speech rhythm develops in L2 English spoken by German learners, and why it is worth studying whether people are sensitive to the changes in L2 speech rhythm, when the target and native languages of the learner are rhythmically similar. A brief overview of empirical studies in German and English speech rhythm is provided to highlight rhythmic similarities between languages. Later, we report the results of the perception experiment aimed to answer the main question of the research: are the rhythmic changes that happen in the course of acquisition perceptually relevant, if the L1 and L2 of the learner are rhythmically similar? In the end, we show the theoretical implications of our findings.

### Speech rhythm and rhythm measures

The word rhythm implies the idea of periodicity. Based on the auditory impression that certain events or certain speech constituents reoccur periodically in the speech stream, the languages were classified into stressed-timed (in which stressed syllables were perceived to be distributed at roughly equal intervals, e.g., German, English, Dutch, Russian) and syllable-timed (in which all syllables were perceived to be of roughly equal duration, e.g., French, Italian, Spanish). Later, a new rhythmic class of mora-timed languages (in which moras are supposedly perceived as roughly equal in duration, e.g., Japanese, West Greenlandic) was added. Experimental studies, however, failed to find empirical evidence to support this impression (Roach, 1982; Dauer, 1983; Pamies Bertran, 1999). However, adults (Ramus et al., 1999) and even infants (Nazzi et al., 1998; Ramus and Mehler, 1999; Nazzi and Ramus, 2003) are able to differentiate between rhythmic patterns of languages that are traditionally classified as stress- and syllable-timed. Therefore, researchers continued looking for the acoustic correlates of auditorily perceived differences in speech rhythm.

A new concept of speech rhythm has been introduced in an attempt to find the perceptually relevant acoustic correlates of rhythmic patterns (Ramus et al., 1999). It rests on the assumption that consonantal and vocalic intervals in the speech signal can exhibit language-specific patterns of durational variability. Languages that are traditionally classified as “stress-timed” exhibit higher degree of durational variability compared to “syllable-timed” languages. That is, stress-timing is characterized by more substantial differences in duration of vowels and consonantal clusters within the same utterance produced by the same speaker. To capture the variability in duration of speech intervals, a number of the so-called rhythm metrics have been proposed. Among the most commonly used interval-based rhythm metrics are the pairwise variability index (PVI) (Grabe and Low, 2002), the standard deviation in duration of speech intervals (Δ) and the percentage of vocalic material in an utterance (%V) (Ramus et al., 1999), the coefficient of variation in duration of speech intervals (Varco) (Dellwo and Wagner, 2003). Conventionally these metrics are applied to vocalic (V) and consonantal (C) intervals, i.e., sequences of consecutive vowels or consonantal clusters that can straddle the syllabic and word boundaries within an utterance). Yet the metrics have also been applied to capture the durational variability of other speech intervals in order to investigate the multiple rhythms on multiple timescale, e.g., on the timescale of feet (Nolan and Asu, 2009) or syllables (Ordin et al., 2011).

Some of these metrics are influenced by the speech rate to a higher degree than the others (Dellwo and Wagner, 2003; Dellwo, 2006; Wiget et al., 2010). For example, ΔV depends on the mean duration of vowels in an utterance. That is, if speech is delivered at a faster rate, mean durations become smaller and ΔV tends to decrease. Dellwo (2006) suggested Varco measure to normalize for the tempo differences and to capture the differences in durational variability irrespective of the differences in speech rate between the languages. Grabe and Low (2002) also suggested a normalized version of PVI in an attempt to neutralize the influence of the speech rate on the measures of the local durational variability. Formulas 1 and 2 show how the raw (rPVI) and the normalized (nPVI) versions are calculated. White and Mattys (2007) and Wiget et al. (2010) reported Varco measures, %V and nPVI are more robust to the fluctuations of the speech rate compared to non-normalized metrics.

(1) $$\text{n }PVI=100\times \left[\sum_{k\;=\;1}^{m\;-\;1}\left|\frac{d_{k}-d_{k\;+\;1}}{(d_{k}+d_{k\;+\;1})/2}\right|/(m-1)\right]$$

(2) $$\text{r }PVI=\left[\sum_{k\;=\;1}^{m\;-\;1}\left|d_{k}-d_{k\;+\;1}\right|/(m-1)\right]$$

where

m—number of interval in an utterance for which PVI is calculated,

d—duration of kth interval.

Higher values of %V and lower values of the other metrics correspond to the languages that are traditionally defined as syllable-timed (Ramus et al., 1999; Low et al., 2000; Grabe and Low, 2002; Dellwo and Wagner, 2003; White and Mattys, 2007), and possibly provide the necessary cues to differentiate the durational patterns of the rhythmically contrastive languages (Ramus and Mehler, 1999; Ramus et al., 1999).

Dauer (1983, 1987) and Schiering (2007) analyzed the phonological structure of languages which give the impression of stress-timing or syllable-timing. Those languages that produce the effect of stress-timing display vowel reduction, more complex C clusters, have more different syllable types, opposition between phonologically long and short vowels, between geminate and non-geminate consonants, are less likely to exhibit vowel harmony and fixed stress. Rhythm metrics reflect these language-specific phonological properties. To name a few examples, ΔC is thought to be indicative of the syllabic structure, syllable complexity and consonantal phonotactic constraints. ΔV is supposed to be indicative of the degree of vowel reduction. VarcoV and VarcoC reflect the same properties ΔC and ΔV do, but Varco measures are supposed to neutralize the effect of the tempo differences, and thus to reduce the effect of idiosyncrasies in speech production. %V indicates the syllabic structure and inventory. Languages with more restricted syllabic inventory operate less complex syllables, usually of the CV structure. The more types of syllables there are in the language inventory, the more consonants are added to the onset or coda of the syllables. This reduces the proportion of vocalic intervals to the overall duration of the utterance, and %V decreases. Prieto et al. (2012) demonstrated that prosodic edges and heads are marked by manipulating durational ratios in a language-specific way, and this may also account for small differences in rhythm measures between languages. The analysis of the surface durational variability of V and C intervals indeed allows spreading the languages on a continuous scale with respect to their rhythmic properties and to say that one language is more or less stress-timed than another, or that the two languages are rhythmically similar. However, it did not yet provide an unambiguous support for the Rhythm Class Hypothesis that suggests that languages are split into distinct rhythm categories.

### Importance of rhythmic patterns for speech processing

People are sensitive to the timing patterns which are captured by rhythm metrics. Mehler et al. (1996) hypothesized that pre-linguistic infants perceive incoming continuous speech as a succession of vocalic and consonantal segments, vocalic segments are processed as informative harmonic signals of variable duration and intensity, which are alternating with unanalyzed noise (consonantal intervals). This Time-Intensity-Grid-Representation of incoming continuous speech is based on innate perception mechanisms that help them to construct the first representation of their language. Ramus et al. (1999) observed that languages with similar rhythmic properties tend to share more typological characteristics of grammatical and phonological structure. This led to the hypothesis that alongside with constructing the first representation of their native language, babies use rhythmic patterns to bootstrap on the syntactic properties of the language and on lexicon (Christophe and Dupoux, 1996; Mazuka, 1996; Mehler et al., 1996, 2004; Nespor et al., 1996). Rhythmic patterns are also used to develop strategies for segmentation of continuous speech and consequent word extraction and learning (Christophe et al., 2003; Thiessen and Saffran, 2007). In light of these considerations, we could suggest that the ability to recognize the durational cues pertaining to the speech rhythm is of the utmost importance for language acquisition and speech processing (e.g., for development and implementation of language-specific segmentation strategies). Therefore, sensitivity to timing differences, which is already observed in infancy, also persists in adulthood (Ramus and Mehler, 1999; White et al., 2012). Adults also use rhythmic cues to recognize the foreign accent in L2 speech and to detect the linguistic origin of the speaker (Kolly and Dellwo, 2014), to evaluate the degree of accentedness in L2 speech (Polyanskaya et al., 2013), to extract discrete linguistic units from continuous speech (Christophe et al., 2003).

### Rhythm changes in second language acquisition

Papers focussed on acquisition of speech rhythm in L2 are rare. Most of these studies concentrate on comparing rhythm in L2 speech with the target represented by an adult native speaker. Examined L2 speech is usually produced by rather advanced learners. The results showed that the rhythm scores in L2 speech are intermediate between those in the native and the target language of the learners (White and Mattys, 2007). This is usually interpreted as the influence of the native language of the learner on his speech production in the L2. Low et al. (2000) showed that nPVI-V in L2 Singaporean English is influenced by the L1 Chinese language. Rhythm in L2 English was shown to be affected by L1 Chinese, French, Spanish, Romanian and Italian (White and Mattys, 2007; Gut, 2009; Mok, 2013, etc.).

The studies with the emphasis on development of rhythmic patterns in the course of L2 acquisition are even rarer. One of the few exceptions is the study by Ordin and Polyanskaya (2014) who compared how speech rhythm develops in L1 and in L2 acquisition. They found that speech rhythm develops from more syllable-timed toward more stress-timed patterns both in child L1 and in adult L2 speech. The authors showed that both vocalic and consonantal variability in duration in L2 English increases as a function of the length of residence in the UK in adult speech when the target (English) and the native (Italian or Punjabi) languages of the learners are rhythmically contrastive.

Ordin et al. (2011) showed that durational variability in speech of L2 learners also increases with proficiency growth when the target (English) and the native (German) languages of the learners exhibit similar rhythmic properties. English and German share phonological parameters that are known to affect the rhythm metrics. Both of these languages are classified as stress-timed in terms phonetic timing patterns captured by metric scores (Grabe and Low, 2002) and exhibit the phonological characteristics typical of stress-timed languages (Dauer, 1987; Schiering, 2007). Therefore German learners of English do not have to acquire phonological characteristics like production of complex syllables and complex consonantal clusters, opposition of long and short vowels, etc. Table 1 provides the metric scores in monolingual adult speech delivered by adult native speakers of either German or English, as reported in various studies. No unambiguous tendency is evident as for in which of these languages the durational variability is higher. %V seems a bit lower in German, which can be explained by a slightly higher syllabic complexity and a higher number of C clusters in German than in English (Delattre, 1965 cited in Gut, 2009). Comparison of the metric scores for German and English with those reported for traditional syllable-timed languages (Ramus et al., 1999; Grabe and Low, 2002; White and Mattys, 2007) shows that both German and English exhibit higher duration variability and lower %V.

**Table 1:** **Metric scores for German and English as reported in various studies**.

**Language**	**Rhythm metrics**						**References**
	**%V**	**VarcoV**	**n-PVI-V**	**rPVI-C**	**ΔC**	**VarcoC**	
German	41.7		52.5	68.7	65.0		Russo and Barry, 2008
	42.8				71.7		Dellwo and Wagner, 2003
	46.4		59.7	55.3	52.6		Grabe and Low, 2002
	39.8	51.5	53.6	67.0	62.0	54.0	Arvaniti (2012)—overall score
	36	44	55	73	62	51	Arvaniti (2012)—scores obtained on read sentences that were deliberately designed to enhance durational variability
	41	52	56	60	54	50	Arvaniti (2012)—scores obtained on read sentences that were deliberately designed to inhibit durational variability
	41	52	53	56	55	50	Arvaniti (2012)—scores obtained on sentences uncontrolled for phonotactics
	42	55	52	72	55	50	Arvaniti (2012)—scores obtained on spontaneous speech
English	38.0	64.0	73.0	70.0	59.0		White and Mattys, 2007
	42.0				55.7		Dellwo and Wagner, 2003
	41.1		57.2	64.1	56.7		Grabe and Low, 2002
	40.1				53.5		Ramus et al., 1999
	45.7	54.8	59.9	68.9	60.0		Arvaniti (2012)—overall score
	41	48	55	83	68	57	Arvaniti (2012)—scores obtained on read sentences that were deliberately designed to enhance durational variability
	50	46	51	57	49	53	Arvaniti (2012)—scores obtained on read sentences that were deliberately designed to inhibit durational variability
	44	50	56	61	55	55	Arvaniti (2012)—scores obtained on sentences uncontrolled for phonotactics
	48	66	66	77	68	59	Arvaniti (2012)—scores obtained on spontaneous speech

### Research question

Previous studies have showed that rhythmic patterns change as language acquisition progresses even when the native and the target languages of the learners are rhythmically similar (Ordin et al., 2011). In our study, we were interested whether these developmental changes in speech rhythm are perceptually relevant. It is already known that the listeners are sensitive to the rhythmic differences between rhythmically contrastive languages (Ramus and Mehler, 1999) as well as between German and English, i.e., between rhythmically similar languages (Vicenik and Sundara, 2013). Listeners are also able to distinguish rhythmic patterns of the utterances from the same language (White et al., 2012; Arvaniti and Rodriquez, 2013). Therefore, we think that the fine distinctions between rhythmic patterns typical of L2 English of adult learners at different proficiency levels might be detected.

These important findings regarding sensitivity of listeners to rhythmic differences have been done using discrimination tests. We know that people can discriminate between utterances even with small differences in durational variability. However, certain functions attributed to speech rhythm are not based on discrimination, but rather on classification (segmentation, evaluation of accentedness, detection of linguistic origin of the speaker, etc.). Classification is different from discrimination. The listener may be able to perceive some acoustic differences when attending to them, but nevertheless ignore these differences when attributing an acoustic signal to a certain group, or when making a decision whether an acoustic signal is a representative of a certain class. In this particular study we focused not merely on whether the differences in L2 rhythm between utterances delivered by learners at different proficiency levels are detected. We were rather interested in whether listeners are able to reliably classify the utterances of L2 learners into distinct classes based on timing differences between utterances, and if so, which timing patterns listeners use to form the classes.

## Speech material

### Participants

Piske et al. (2001) analyzed a range of factors that influence pronunciation of L2 learners. These factors, among others, included the age and the length of exposure to the L2, amount of L2 use, language learning aptitude and motivation, learning mode. In our study we controlled these factors by collecting the relevant information in a detailed language-background questionnaire (see Appendix 1 in online Supplementary Materials). Based on the questionnaire, we selected only those speakers who formed a homogeneous group and varied only in the degree of L2 mastery. The relevant information gleaned from the questionnaire was further verified in an informal interview during the recording sessions.

We have recorded 51 German learners of L2 English (17–35 years old, *M* = 21; 27 females). We selected for participation only those people who grew up in or near the city of Bielefeld in North-Rhein Westphalia. The variety of German spoken in that region closely resembles what is understood as a Northern standard variety of German (Hochdeutsch). The selected participants did not exhibit features of regional varieties of German. All the participants were monolingual native speakers of German without speech or hearing disorders.

We have also recorded 10 native speakers of English (southern British variety, 25–40 years, *M* = 30, 6 females) to compare the metric scores of the L2 learners of English with those of the L1 English speakers. The English speakers were residents in Germany at the time of the recordings. However, they reported to have little to no command of German, lived in close English-speaking community at the UK military bases in Nord-Rhein Westphalia, worked in only English-speaking environment, had English as their home and neighborhood language, came from monolingual English-speaking families and were raised in monolingual environment.

### Elicitation procedure

The selected learners of English first underwent a pronunciation test so that we could assess the learners' mastery of pronunciation. The test was devised by the authors and consisted of two parts: Perception and production. The perception part was compiled from Vaughan-Rees (2002) and included phoneme recognition, emotion recognition, intention recognition tasks. The production part included sentence reading. The sentences for production were composed to evaluate segmental realizations and prosodic control of the participants in the second language. The test ran for approximately 20 min. The test and the details on the controlled pronunciation features and assessment criteria can be found in Appendix 2 in online Supplementary Materials.

Further on, a 5-min phonetic aptitude test (PAT) was administered. The authors devised this test based on the oral mimicry tests described by Pike (1959), Suter (1976), and Thompson (1991). The test is aimed to predict the general phonetic ability by asking the participant to imitate novel sounds that do not exist in their native or target language and to mimic novel prosodic phenomena (e.g., lexical tones, tonal contours with accents which are not aligned according to the convention of the learner's target or native language, etc.). The test and the details on the assessment criteria can be found in Appendix 3 in online Supplementary Materials. The sounds to imitate were presented by the holder of the IPA certificate confirming his proficiency in producing and perception of sounds existing in world languages. The performance of participants in PAT did not correlate with their L2 proficiency (we had both high and low proficiency learners with both high and low phonetic aptitude). Neither did the performance of the L2 learners in the PAT correlate with their performance in the English pronunciation test with any of the metrics calculated on their speech. This shows that the ability to imitate rhythmic patterns of the target language is not related to the general phonetic aptitude and we can eliminate a potential alternative explanation that the differences in rhythmic patterns between learners at different proficiency levels are pertaining to the phonetic aptitude rather than to the overall proficiency.

At the next stage, an informal interview was conducted by the first author. General questions about preferences in reading and music, lifestyle, career choice, biography, and childhood were asked (Appendix 4 in online Supplementary Materials). The interviews were recorded and lasted approximately 12 min long with each participant.

Following the interview, we ran a sentence elicitation task, similar to one used by Bunta and Ingram (2007). Thirty three sentences were elicited from each speaker. We used 33 picture prompts for the elicitation procedure. The participants viewed picture slides in PowerPoint presentation. Each slide was accompanied with a descriptive sentence. The participants were instructed to remember the sentences. The participants could move to the next image or to go back to the previous slide at their own pace. When they had viewed all the slides, they were asked to look at the images again, without the accompanying text, and to recall and say the sentences that they had been asked to remember. In a very rare case (<5%) when the speaker could not remember the sentence or retrieved a modified sentence from memory, verbal prompts were used to help the speaker to produce the correct sentence. For example, the participant said “The dog is running after the cat,” and the expected sentence was “The dog is chasing the cat.” The researcher responded to the participant: “Yes, it is. You could also say *chasing*, which means *running after*. Can you say what you see at this picture once again?” One verbal prompt was sufficient to elicit the expected sentence when there was a mismatch in the first trial. The recording ran continuously throughout the sentence elicitation procedure. The list of elicited sentences and the examples of picture prompts can be found in Appendix 5 in online Supplementary Materials.

The tests and recordings were made individually with every participant in a sound-treated booth of the audio-visual studio at the Bielefeld University in Germany. The recordings were made in WAV PCM at 44 kHz, 16 bit, mono.

### Assessment of learners' proficiency

Three experienced teachers of English as a foreign language listened to the recorded interviews and evaluated learners' fluency, grammatical accuracy, and vocabulary resources. They used a 10-point scale for each parameter. To estimate the consistency of ratings between the teachers, we used Cronbach alpha, which is 0.90 for vocabulary, 0.89 for fluency and 0.92 for grammatical accuracy. This shows high agreement between the raters and confirms the reliability of their assessments. We averaged three ratings across the parameters for each rater and each interview, and thus got three mean ratings per learner.

The teachers' assessments and the results of the pronunciation tests were used to place the learner into one of the following proficiency groups: beginners (12 speakers with ratings between 4 and 6), intermediate (9 speakers with mean ratings between 6 and 8), and advanced learners (22 speakers with ratings above 8)^1^. We used the results of the pronunciation test to assess the pronunciation skills of the learners. Eight speakers were not attributed to any group, either because the teachers did not agree with each other in their assessments (2 speakers were excluded for this reason) or because of the discrepancy between the results of the pronunciation tests and the teachers' assessment of accuracy, fluency and vocabulary resources. Pronunciation skills do not always agree with the general assessment of the learner's reading, writing, listening and speaking skills, vocabulary size, grammar accuracy, etc. That is why we deemed it necessary to combine the tutors' assessment of fluency, accuracy and vocabulary on the one hand and the mastery of pronunciation on the other hand. In case when pronunciation lags far behind the general L2 mastery or exceeds the expected level, the learner was not attributed to any of the proficiency groups.

### Segmentation

Thirty three elicited sentences per speaker were annotated in Praat (Boersma and Weenink, 2010). Annotation was performed by the second author. Each sentence was divided into V and C intervals. The segmentation was carried out manually by the second author based on the criteria outlined in Peterson and Lehiste (1960) and Stevens (2002) for V and C intervals.

The burst of energy corresponding to the release of the closure was taken as the starting point of a consonantal interval with the initial voiceless plosive sound after a pause and at the beginning of a sentence. Either the stop release, or apparent beginning of a voice bar, or other cues indicating apparent vibration of the vocal folds (whatever came first) were considered as the beginning of a consonantal interval with the initial voiced plosive. The markers of the turbulent noise were taken as the beginning of fricative consonants. The beginning of the first formant was taken as the beginning of a sonorant consonant. Consonantal intervals in the middle of a sentence were considered to start after the vowel finishes, and to stretch until the onset of the following vowel. The end of the consonantal interval in the final position was marked at the end of the acoustic energy. The consonantal intervals in the final positions were considered to start immediately after the vowel and finish at the end of the fricative noise (for obstruents) or at the end of the first formant (for sonorants). Conventional procedure based on the analysis of the waveform and the spectral characteristics of the speech signal was based to identify the boundaries of the vocalic intervals. The end of the vowel was identified by the abrupt change in the vowel formant structure or by termination of the formants, and by the significant drop in the waveform amplitude. The onset of the vowel was marked at the beginning of the voicing identified as the start of the regular vertical stripes on the spectrogram in the region of the second and higher formats. The marker indicating the vowel onset or offset was placed at the point closest to the zero crossing on the waveform.

In difficult cases where it was necessary to place the boundary between the consonantal interval represented by a sonorant consonant with a clear formant structure and a vowel, the decision was based on the amplitude of the first format. Such difficult cases were associated with the boundaries or categorizing allophones of /l/ (e.g., in the words *girl*, *ball*, *table*). We based our segmentation on purely phonetic criteria, therefore /l/ was sometimes marked as a vowel (in case of a vocalized [l]), and sometimes as a consonant. The decision was based on (1) auditory analysis by an experienced phonetician, and (2) amplitude of the first formant. If the amplitude did not drop after the preceding vowel and the segment was perceived by a phonetician as a vocalized [l], then the segment was segmented as a vocalic interval. We did not want to pre-define certain types of segments either as consonantal or vocalic. We adopted a phontic approach to speech rhythm. Within the adopted framework, speech rhythm is represented by the surface timing patterns, which are purely phonetic, and phonetic properties are not discrete and cannot be pre-assigned to a certain phonological category a-priory.

Pauses and hesitations were not included into V or C intervals and were discarded. If the same type of the interval was annotated prior and following the pause, we treated them as two separate intervals because they are likely to be perceived as such. Final syllables were included into analysis.

### Calculating the rhythm metrics

The sentences elicited using the picture prompts were used to calculate the rhythm metrics. The sentence elicitation procedure helped us to avoid the reading mode and made speech material more similar to natural spontaneous speech. Besides, we obtained lexically identical sentences from every participant, which is necessary to analyze the development of speech rhythm *per se*, not affected by the differences between the sentences in phonotactics, number of syllables in polysyllabic words, syntactic structures and phrasing.

Traditional rhythm metrics were calculated on each sentence. The overview of the selected metrics was given in the Table 2. We also calculated the mean duration of V and C intervals for each sentence to account for possible developmental changes in speech tempo in the course of L2 acquisition, and for the interaction of speech rhythm and speech tempo.

**Table 2:** **Metrics used in this study**.

**Metric**	**Description**
%V	Percentage of vocalic intervals
ΔV	Standard deviation of vocalic intervals duration
ΔC	Standard deviation of consonantal intervals duration
nPVI-V	Averaged of the mean differences between successive vocalic intervals
nPVI-C	Averaged of the mean differences between successive consonantal intervals
rPVI-V	Averaged difference in duration of successive vocalic intervals
rPVI-C	Averaged difference in duration of successive consonantal intervals
VarcoV	Coefficient of variation of vocalic intervals, i.e., standard deviation divided by the mean
VarcoC	Coefficient of variation of consonantal intervals, i.e., standard deviation divided by the mean
MeanV	Mean duration of vocalic intervals
MeanC	Mean duration of consonantal intervals

Although some rhythm metrics were claimed to be better than others at quantifying rhythm, there is no consensus on which metrics have more discriminative power. White and Mattys (2007), for example, advocated for pairwise metrics, while Ramus et al. (1999) favored ΔC and %V. Loukina et al. (2011) performed the analysis of 15 rhythm metrics and in experiments separating pairs of languages by rhythmic properties showed that a rhythm measure that is successful at separating one pair often performs poorly at separating another pair. Considering the lack of consensus on the optimal set of metrics, we decided not to limit our investigation to the metrics which were found more useful in certain studies. Instead we tested all the metrics in order to see which ones better capture the differences in rhythm between sentences produced by L2 learners at different proficiency levels.

A series of by-sentence ANOVA tests (Table 3) with the *values of the metrics* as the dependent variables and *proficiency level* as the factor shows that non-normalized rhythm metrics (ΔV, ΔC, rPVI-v, rPVI-c) and %V do not differ between the proficiency levels. As the raw metrics do not differ between the proficiency levels, we are not including them into further statistical tests.

**Table 3:** **Non-significant ANOVA tests for the rhythm metrics between proficiency groups**.

**Metric**	**Significance of levene's test**	**Significance of welch test (if Levene's test is significant) or *F* statistics of the analysis of variance**
rPVI-v	0.513	0.4
rPVI-c	0.007	0.267
ΔV	0.748	0.692
ΔC	<0.0005	0.154
%V	0.691	0.068

**Figure 1:**
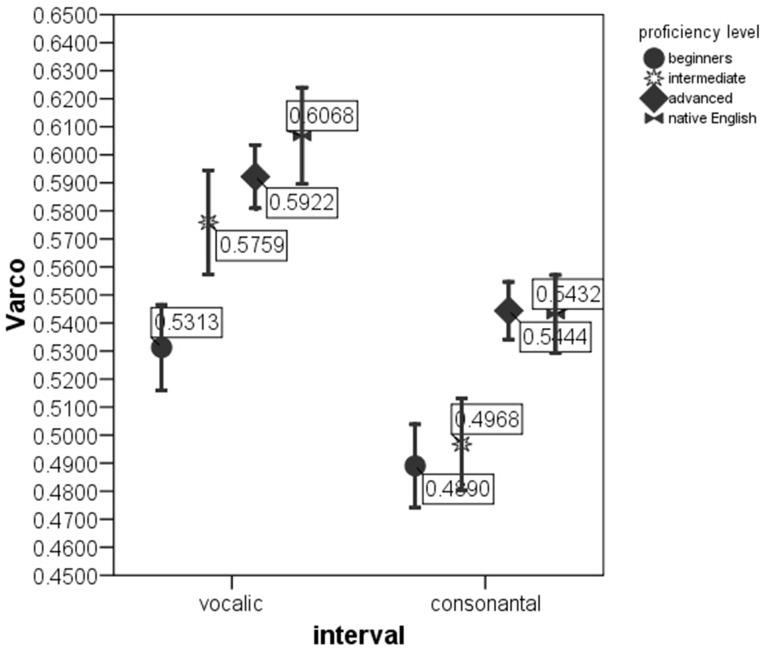
**VarcoV and VarcoC in the sentences produced by native English speakers and by German learners of English at beginning, intermediate and advanced proficiency levels**. Error bar shows 95% confidence interval.

**Figure 2:**
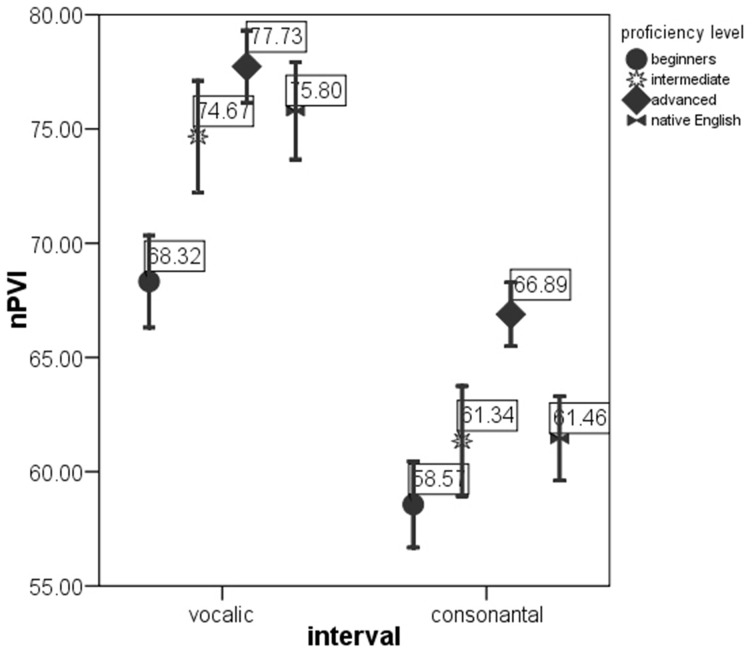
**nPVI-v and nPVI-r in the sentences produced by native English speakers and by German learners of English at beginning, intermediate and advanced proficiency levels**. Error bar shows 95% confidence interval.

**Figure 3:**
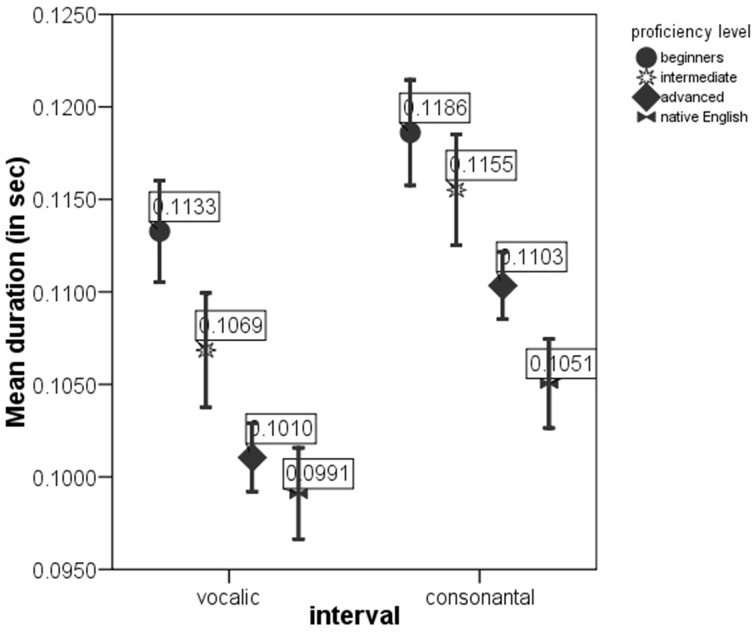
**meanV and meanC in the sentences produced by native English speakers and by German learners of English at beginning, intermediate and advanced proficiency levels**. Error bar shows 95% confidence interval.

ANOVAs on the rate-normalized rhythm measures revealed significant difference between *proficiency levels* at *p* < 0.0005 for each metric. These metrics were included into multivariate model. The MANOVA test with *nPVI metrics, Varco metrics* and *mean* durations of V and C intervals as the dependent variables and *proficiency level* as the factor revealed a significant effect of *proficiency level* on the rhythm measures, Λ = 0.856, *F*_(12, 2822)_ = 19.06, *p* < 0.0005, μ^2^ = 0.075. Figures 1–3 show that the metric scores increase as L2 acquisition progresses, which indicates that German learners of English deliver L2 speech at a higher rate and with higher degree of stress-timing as their L2 mastery grows. The differences between the proficiency levels pairwise for each metric are mostly significant (significance values are given in Table 4).

**Table 4:** **Significance for comparisons of rhythm metrics between proficiency levels pairwise (with Hochberg's correction)**.

**Comparison**	**VarcoV**	**VarcoC**	**nPVI-V**	**nPVI-C**	**meanV**	**meanC**
Beginner—Intermediate	<0.0005	0.855	<0.0005	0.17	0.004	0.315
Intermediate—Advanced	0.328	<0.0005	0.096	<0.0005	0.004	0.011

**Figure 4:**
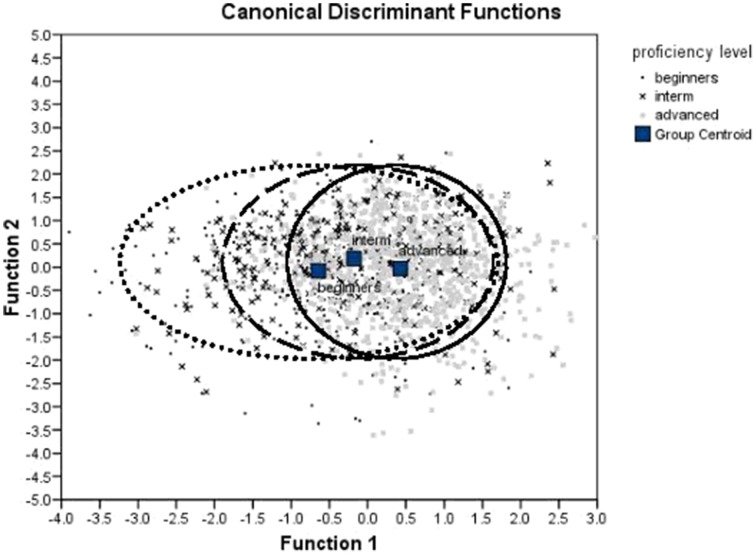
**Discriminant function plot**.

The MANOVA was followed up with the discriminant analysis. We used only those metrics that were found to differ significantly between proficiency levels in our previous tests. The analysis revealed two discriminant functions. The first function explained 96.9% of variance, canonical *R*^2^ = 0.14, and the second explained only 3.1% of variance, *R*^2^ = 0.005. In combination these functions significantly differentiated the proficiency levels, Λ = 0.856, χ ^2^_(12)_ = 220.318, *p* < 0.005. The second function alone did not significantly differentiate between the proficiency levels, Λ = 0.995, χ ^2^_(5)_ = 7.232, *p* = 0.204. This can also be seen on the discriminant function plot (Figure 4). Classification results (Table 5) show that the model classifies correctly 57% of cases (chance is 33%).

**Table 5:** **Classification Results based on the Discriminant Analysis**.

		**Predicted Group Membership (in %)**		
		**Beginners**	**Intermediate**	**Advanced**
Original Group	Beginners	38.4	0	55.1
Membership (in%)	Intermediate	26.6	0.3	73.1
	Advanced	9.8	0	90.2

The correlations between the outcomes and discriminant functions revealed that the measures of local—pairwise—variability and of speech rate loaded on the first function, and global measures of variability loaded more highly on the second function (see Table 6). As the first function explains substantially more variance that the second function, we can conclude that the pairwise durational variability and speech rate discriminate between the proficiency levels much better than utterance-wise variability.

**Table 6:** **Structure matrix of the discriminant function coefficients**.

	**Function**	
	**1**	**2**
meanV	−0.5^**^	0.07
nPVI-C	−0.472^**^	−0.404
nPVI-V	−0.469^**^	−0.444
meanC	−0.347^**^	−0.224
VarcoC	0.436	0.79^**^
VarcoV	0.409	−0.552^**^

*The stars indicate larger correlation between each variable and one of the discriminant functions*.

We also wanted to see how close the advanced German learners of English are to their target in regard to acquisition of rhythmic patterns. For this, we compared the metric scores calculated on the sentences produced by the advanced learners of English with those calculated on the sentences spoken by native English speakers. *T*-tests (Table 7) reveal that the metric scores do not differ between sentences spoken by advanced German learners and native speakers of English, with the exception of meanC (overall shorter C intervals in the utterances of L2 speakers) and rPVI-C (raw pairwise variability of consonantal intervals is higher in speech of learners of English). The difference in ΔC is on the verge of significance (*p* = 0.069), and the scores are again higher in sentences produced by L2 learners. Significant and marginally significant difference in consonantal variability is easily accounted for the differences in articulation rate of C intervals: longer C intervals in L2 speech result in larger standard deviations and pairwise durational differences. What is important is that pairwise durational variability of consonantal intervals *per se*, i.e., when the differences in speech rate are normalized, is also significantly higher in speech of advanced L2 learners. Advanced learners overshoot with increasing durational variability of C intervals, although they successfully acquire variability of V intervals. This can be explained by less assimilation of consonants in clusters within syllables in L2 speech (i.e., tendency to clearly produce all the consonants in the clusters) and by incomplete mastery of fine modifications in prosodic timing for the purposes of marking edges and heads of prosodic constituents (see Prieto et al., 2012).

**Table 7:** ***t*-tests comparing metric scores in English speech of native English speakers and advanced L2 learner of English**.

	**meanV**	**meanC**	**%V**	**ΔV**	**ΔC**	**VarcoV**	**VarcoC**	**rPVI-V**	**rPVI-C**	**nPVI-V**	**nPVI-C**
*t*_(1054)_	1.216	3.371	−1.314	−0.064	1.818	−1.433	0.131	1.014	4.506	1.412	4.506
*p*	=0.224	=0.001	=0.189	=0.949	=0.069	=0.152	=0.896	=0.311	<0.0005	=0.158	<0.0005

The analysis shows that speech rate and the degree of stress-timing increase as a function of proficiency growth. This tendency, however, can only be captured by normalized rhythm metrics. Raw metrics do not differ between the proficiency levels. The values of the raw metrics are influenced by the speech tempo, i.e., by the mean durations of speech intervals: The faster one talks, the shorter the V and S intervals become; the shorter speech intervals result in smaller durational differences in pairs of consecutive intervals and in smaller standard deviation in duration of speech intervals. As the mean durations of speech intervals significantly differ between the proficiency levels, we should also expect significant differences in the values of the raw metrics. However, this was not confirmed. We believe that the values of the raw metrics are influenced by two conflicting forces: The tendency to deliver speech at a faster rate and with higher durational variability at high proficiency levels. This conflict prevents the emergence of significant differences in the values of raw metrics between proficiency levels. Normalization—removing the influence of speech tempo—allows us to notice the trend to enhance durational variability in L2 speech with proficiency. The lack of significant differences in %V between the proficiency levels presents an interesting case. In earlier studies %V has been reported to be robust to fluctuations in speech tempo (Wiget et al., 2010) and to discriminate between L1 and L2 speech (White and Mattys, 2007). However, in our experiment %V was not informative. %V is the proportion of the vocalic material in a sentence, and that is determined by phonotatic differences. The proportion of vocalic material will be lower in the languages that allow complex consonantal clusters and reduction of vowels in unstressed positions (e.g., German, Russian, English). These languages are traditionally classified as stress-timed (Dauer, 1983). The languages on the opposite end of the spectrum impose strong phonotactic constraints, prefer simple CV syllables and feature less vowel shortening (e.g., French, Japanese). These factors increase the proportion of vocalic material in speech. Therefore, we assume that %V is a powerful predictor to discriminate between rhythmically contrastive languages. %V can also reflect the differences in lexical material, i.e., whether the utterances *per se* differ in phonotactic characteristics. In our study, we used the same set of sentences elicited from different speakers, thus the lexical differences that could potentially influence %V were eliminated. The target and the native languages of the L2 learners were similar in terms of phonotactic and phonological properties, and the learners did not have problems with producing the clusters of consonants in English sentences. %V captures phonotactic and phonological differences, but the sentences spoken by learners at different proficiency levels in our study manifested only phonetic differences in timing patterns, phonotactics and phonological characteristics were the same. Therefore, it is not surprising that %V was not found to differ between sentences produced by L2 learners at different proficiency levels.

The discriminant analysis also reveals that the advanced learners are more consistent in realization of timing patterns compared to lower-proficient learners. Inspection of the discriminant function plot (**Figure 7**) reveals that the variate scores for the advanced learners are more compact, while the variate scores for the beginners are spread more evenly along the first discriminant function. The discriminant function plot also showed that the variate scores for different groups of acquirers overlap (see overlapping circles on **Figure 7**). This means that beginners produced sentences sometimes with high degree of durational variability, and sometimes with lower degree of durational variability. Advanced learners constantly produced the sentences with high degree of durational variability. In other words, the productions of beginners varied greatly between stress-timed and syllable-timed rhythm patterns, but productions of advanced learners were more consistently stress-timed.

We can draw the same conclusion if we look at Table 5. Rhythm and tempo measures correctly predict the speaker's proficiency level for 57% of sentences. The overall accuracy is significantly above chance (33%), but the accuracy for the sentences produced by speakers on different proficiency levels varies substantially. Sentences produced by advanced speakers were classified correctly in 90.2% of cases, while sentences produced by beginners were classified correctly only in 38.4% of cases. This means that the half of the sentences spoken by beginners exhibit higher degree of variability that is typical of stress-timed rhythm in 90.2% sentences spoken by advanced learners. On the other hand, only 9.8% of sentences spoken by advanced learners exhibit lower durational variability overlapping with 38.4% of sentences from beginners. The analysis of the discriminant function plot and the classification accuracy indicates that the timing patterns become more stable and consistent as a result of the acquisition progress.

To conclude, the analysis confirms significant differences in rhythmic patterns between proficiency levels in L2. Rhythm measures are more consistently stress-timed at higher proficiency levels. Raw metrics are influenced by conflicting tendencies to deliver speech at a faster rate and with higher durational variability at higher proficiency levels, and thus do not increase with proficiency. The developmental tendency to increase the degree of stress-timing in L2 speech has been observed even when both the native and the target languages of the learner are rhythmically similar. The main research question of our study was to investigate the perceptual relevance of the rhythmic differences between proficiency levels. Based on the literature review, we assumed that listeners are sufficiently sensitive to the durational variability of C and V intervals to discriminate timing patterns of L2 utterances delivered by learners at different proficiency levels. We wanted to find out whether the detected differences in timing patterns between proficiency levels are used to classify utterances into discrete categories. To address this question, we set up the perception experiment.

## Experiment

### Methods

#### Participants

We have recruited 25 native English speakers to act as listeners in the perception study (age range—21–24 years, *M* = 22; 13 females). Care was taken to form a socially homogeneous group of listeners with the same language background. All participants were students of Ulster University, monolingual English speakers (see our criteria for monolinguality in the description of the participants for Experiment 1). All listeners grew up in or around Belfast and were speaking the same regional variety of English (verified by a native speaker of English, phonetician and Belfast resident). We ensured that the participants did not differ in age, educational level, social status, language background, experience with foreign languages, and all had equal exposure to educated standard British English.

#### Stimuli

We selected sentences elicited from seven speakers per proficiency group in the first experiment to prepare the stimuli. The selected speakers from the advanced group had the highest mean ratings given by the evaluators (see description of the first experiment, Section *Procedure*). The selected speakers from the beginners had the lowest mean ratings from the evaluators. We also randomly selected seven speakers from the group of intermediate learners.

Eighteen out of thirty tree elicited sentences per speaker were selected for stimuli preparation. Six sentences had three stressed syllables (e.g., *the ‘dog is ‘ eating the ‘bone*), six sentences included two stressed syllables (e.g., *the ‘book is on the ‘table*) and six sentences had only one stressed syllable (e.g., *it's ‘raining outside*). The selected sentences produced by the selected speakers were listened to in order to make sure that the sentences were indeed pronounced with the expected number of stressed syllables. The selected sentences are marked with asterisk in Appendix 5 in online Supplementary Materials. We selected 378 sentences in total for the perception experiment (21 speakers ^*^ 18 sentences).

We used the speech resynthesis technique (Ramus and Mehler, 1999) to prepare the stimuli. We replaced all consonantal intervals in the selected sentences with “*s”* and all vocalic intervals with “*a”* and resynthesizing sentences with constant fundamental frequency in MBROLA. The durations of “*s”* and *“a”* intervals were equal to the duration of C and V intervals in the original sentences. This technique degraded segmental and most of the prosodic information from the sentences. The only preserved differences between the identical sentences spoken by learners at different proficiency levels were the differences in durational ratios of C and V intervals. Regardless of the recent criticism of this technique (Arvaniti and Rodriquez, 2013), its usefulness has been demonstrated in a number of studies (Ramus et al., 1999; Ramus and Mehler, 1999; Vicenik and Sundara, 2013; Kolly and Dellwo, 2014, etc.), and we found this delexicalization method to be optimal for the purposes of our study.

#### Procedure

The experiment was carried out with each participant individually in the phonetic laboratory of Ulster University. The stimuli were presented to the listeners in two sessions: Training and testing. The listeners were not informed that the stimuli were derived from L2 English speech because we did not want the listeners use linguistic expectations regarding what the stimuli in L2 English might sound like. This might have created a bias that would be difficult to control. Instead, the listeners were told that the stimuli were derived from three rare exotic African languages. We coined these languages *Burabah* (sentences of the advanced L2 learners converted into “*sasasa”* stimuli), *Losto* (stimuli based on durations in sentences of intermediate learners of English), and *Mahutu* (resynthesized sentences of beginners).

We chose 108 stimuli for the training session (18 stimuli per speaker, 2 speakers per proficiency group). Before the session, each listener was exposed to nine stimuli, randomly selected from those used later in the training session, 3 stimuli per proficiency group, i.e., per “exotic language.” The listener had 1 min to listen to these stimuli by clicking with a mouse on nine buttons on the computer screen. Each button had a caption with the “language” name. After 1-min familiarization, the stimuli were presented to listener one by one. The listener had to identify from which language (Mahutu, Losto, or Burabah) it originates. The listener was expected to click one of the three buttons on the computer screen with a mouse pointer. Each button had a caption with the “language” name. On response, the listener was provided with the feedback which “language” it really was, and the next stimulus was played. When all 108 stimuli were presented, the participant had a 2-min break before the stimuli were played again. The training procedure was repeated three times. Supposedly, during the training session the participants formed new perception categories for further discrimination between the stimuli from different groups. Then the testing session began.

For the testing session, we prepared 270 stimuli (different from those used in the training session, 5 speakers per proficiency group, 18 sentences per speaker). The procedure was the same as in the training session, but the listeners received no feedback, and all the stimuli were played only once.

The duration of the experiment varied between participants and usually exceeded 90 min. The participants could take a short break and have a rest pause during the training session and between the training and the testing session, but not during the testing session. During the experiment the participants were offered hot and cold drinks and sweet snacks to help them cope with possible fatigue. The participants could have their drinks and snacks during the rest pauses as well as during the training session. The order of stimuli presentation was randomized using the internal Praat algorithm in attempt to counterbalance for possible fatigue effect.

## Results

We calculated rhythm metrics on the stimuli that were classified by the majority of listeners as Burabah, Losto, and Mahutu. The metrics were calculated on V and C intervals. We performed the discriminant analysis to test whether rhythm metrics statistically discriminate between the stimuli classified into three groups. The analysis revealed two discriminant functions. The first function explained 94.8% of variance, *R*^2^ = 0.52, and the second function explains 5.2% of variance, *R*^2^ = 0.05. These functions in combination significantly differentiate between the groups, λ = 0.457, χ ^2^_(20)_ = 149.9, *p* < 0.0005. The second function alone is not significant, λ = 0.945, χ ^2^_(9)_ = 11.101, *p* = 0.282. The overall accuracy of the model is 69% (chance is 33.3%), accuracy of Burabah is 91%, Losto—52%, Mahutu—58.5% (chance level is 33.3% for each category). See Table 8 for the details on the classification accuracy.

**Table 8:**
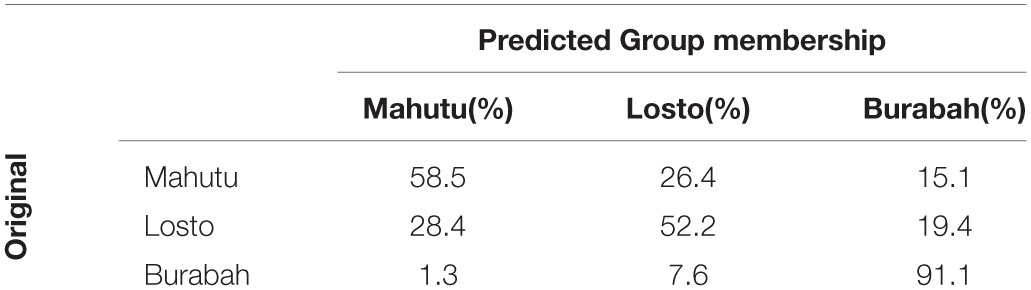
**Classification Results (prior probabilities: all groups equal)**.

The structure matrix (Table 9) reveals that the first function is loaded with the raw metrics and mean durations of V and C intervals, while the second function is loaded with the normalized metrics. This means that the normalized metrics cannot discriminate between the groups, but mean durations and raw metrics discriminate between the stimuli identified as Burabah, Losto, and Mahutu with probability significantly above chance.

**Table 9:** **Structure matrix of the discriminant function coefficients**.

**Metrics**	**Function**	
	**I**	**II**
meanV	0.719^*^	0.404
meanC	0.607^*^	−0.181
rPVI-v	0.458^*^	0.351
rPVI-c	0.371^*^	−0.097
ΔC	0.352^*^	−0.064
ΔV	0.421	0.559
VarcoV	−0.002	0.341^*^
nPVI-c	0.102	0.208^*^
nPVI-v	0.002	0.203^*^
VarcoC	0.035	0.127^*^

*The stars indicate larger correlation between each variable and one of the discriminant functions*.

**Figure 5:**
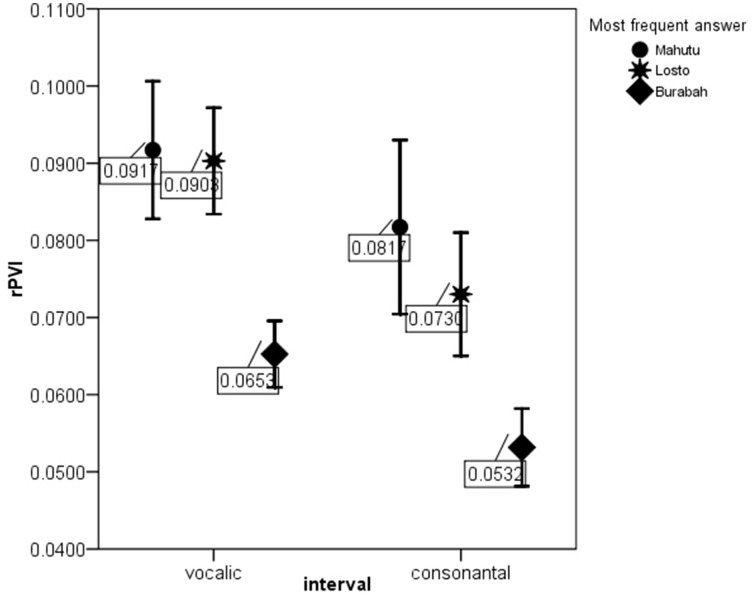
**rPVI-V and rPVI-C for the stimuli identified as Mahutu, Losto, or Burabah**. Error bar shows 95% confidence interval.

**Figure 6:**
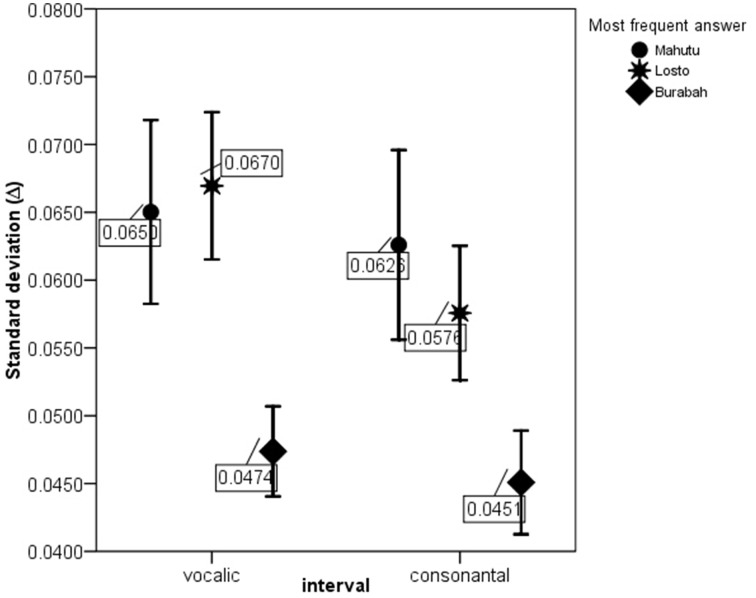
**ΔV and ΔC for the stimuli identified as Mahutu, Losto, or Burabah**. Error bar shows 95% confidence interval.

**Figure 7:**
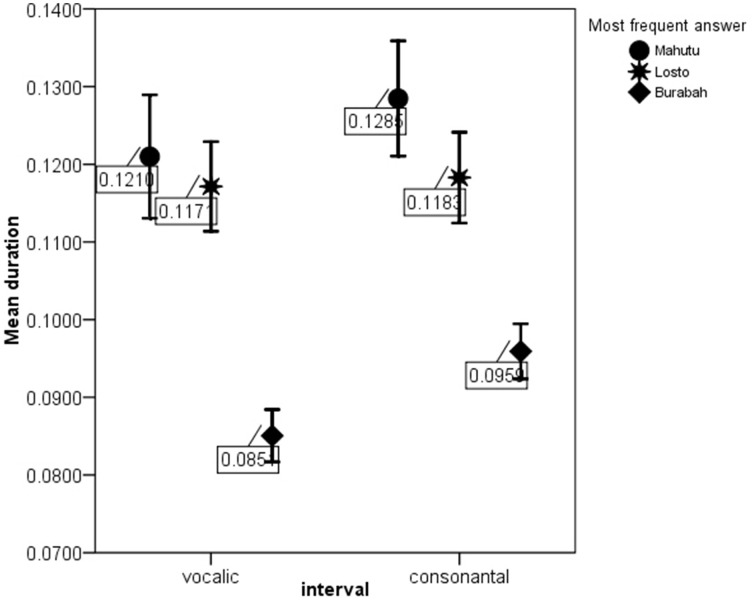
**meanV and meanC for the stimuli identified as Mahutu, Losto, or Burabah**. Error bar shows 95% confidence interval.

Figures 5–7 show the differences in the rhythm metrics that significantly differ between stimuli classified into three groups. Only mean durations and non-normalized metrics (rPVI and the standard deviation) differ significantly between the stimuli identifyed as Burabah, Mahutu, and Losto and statistically discriminate between the groups. Rhythm metrics normalized for tempo and %V do not differ between the stimuli classified into three different groups, and do not discriminate between stimuli attributed to different classes.

Figures 5–7 show that the stimuli identified as Burabah exhibit shorter mean durations and smaller standard deviations in duration of speech intervals and smaller durational differences in pairs of consecutive intervals. As the raw metrics are influenced by the speech rate, we cannot say what the participants were listening for to make their judgments—speech tempo, durational variability, or both. As meanV and meanC display the highest correlations in the structure matrix (Table 9), we could conclude that tempo is *probably* more important than durational variability for the listeners performing the classification task. However, we still do not know the relative contribution of the rhythm compared to tempo in classification. To address this issue, we calculated the frequency for Burabah, Losto, and Mahutu response for each stimulus, i.e., how many listeners out of 25 identified each stimulus as Burabah (*Frequency_Burabah*), Losto (*Frequency_Losto*), or Mahutu (*Frequency_Burabah*). After that we performed stepwise multiple regression to assess the ability of *meanV, meanC, rPVI-C, rPVI-V, ΔV*, and *ΔC* to predict *Frequency_Burabah*. Stepwise regression was chosen because we wanted to evaluate whether both the mean durations and the variability measures were necessary to predict *Frequency_Burabah*. The constructed model included only two steps. At the first step, *meanV* was entered into equation as the most powerful predictor. At the second step, *meanC* was added, and the model was significantly improved. Adding raw rhythm metrics as predictors did not improve the model further. Table 10 summarized the main details of the regression model.

**Table 10:** **Coefficients and parameters of the regression model with Frequency_Burabah as the dependent variable**.

**Step**	**Metrics**	**β**	***t***	***B***	***p***	***R*^2^**	***R*^2^ change**	**Significance of *R*^2^ change**
1	meanV	−0.638	−13.574	−102.93	<0.0005	0.407	0.407	<0.0005
2	meanV	−0.489	−10.506	−78.832	<0.0005	0.519	0.111	<0.0005
	meanC	−0.365	−7.853	−64.49	<0.0005			

The results show that the most important predictors are mean durations of V and C intervals, which are negatively correlated with the frequency of “Burabah” response. This means that the shorter the speech intervals (i.e., the faster the tempo), the more likely the listener will classify the stimulus as Burabah.

We also performed stepwise multiple regressions with *Frequency_Mahutu* and with *Frequency_Losto* as dependent variable (details of the regression models are in Tables 11, 12 respectively). The analyses show that the most influential predictors for both *Frequency_Mahutu* and *Frequency_Losto* are *meanV* and *meanC*. The predictors are positively correlated with the frequency of “Losto” and “Mahutu” responses, which means that the stimuli with longer C and V intervals (i.e., slower speech rate) are more likely to be identified as Losto or Mahutu.

**Table 11:** **Coefficients and parameters of the regression model with Frequency_Mahutu as the dependent variable**.

**Step**	**Metrics**	**β**	***T***	***B***	***p***	***R*^2^**	***R*^2^ change**	**Significance of *R*^2^ change**
1	meanV	0.525	10.1	61.108	<0.0005	0.276	0.276	<0.0005
2	meanV	0.377	7.163	43.814	<0.0005	0.386	0.110	<0.0005
	meanC	0.363	6.914	46.28	<0.0005			

**Table 12:** **Coefficients and parameters of the regression model with Frequency_Losto as the dependent variable**.

**Step**	**Metrics**	**β**	***t***	***B***	***p***	***R*^2^**	***R*^2^ change**	**Significance of *R*^2^ change**
1	meanV	0.427	7.741	41.821	<0.0005	0.183	0.183	<0.0005
2	meanV	0.358	5.993	35.018	<0.0005	0.207	0.024	=0.005
	meanC	0.170	2.847	18.207	=0.005			

## Discussion

The results show that listeners classify the stimuli based on speech tempo and ignore the differences in the durational variability between the “*sasasa”* sequences. The Figures 5–7 also show that there is no difference between the stimuli identified as Losto and Mahutu for ΔV, ΔC, rPVI-V, rPVI-C, meanC, and meanV measures. Faster stimuli with both low and high variation in duration of V and C intervals were classified as Burabah, and slower stimuli were almost randomly attributed to either Losto or Mahutu. We conclude that the listeners formed only two categories: one for faster stimuli that were classified as Burabah, and the other for slower stimuli that were randomly identified either as Mahutu or Losto.

This result agrees with psychoacoustic data in tempo perception. Quene (2007) and Thomas (2007) studied just-noticeable differences in tempo and found that 5–8% change in tempo (expressed as beats per minute for non-speech stimuli and syllables-per-minute for speech stimuli) is easily detected by the subjects. We analyzed the tempo differences between the stimuli which were classified as Losto, Mahutu, and Burabah. Average tempo equals 5.62 syl/s. for the stimuli identified as Burabah, 4.41 syl/s. for the stimuli identified as Losto, and 4.4 syl/s. for the stimuli identified as Mahutu. ANOVA analysis showed that the difference in tempo between the groups is significant, *F*_(2, 196)_ = 64.077, *p* < 0.0005. Pairwise comparisons (with the Bonferroni correction) reveal that the difference lies between “Losto” and “Burabah” stimuli, while the difference between “Losto” and “Mahutu” groups is not significant. Speech tempo in the stimuli identified as Burabah is 25.7% higher than in the stimuli identified as Losto. This increase is above the threshold for just noticeable tempo difference (Quene, 2007; Thomas, 2007). Speech tempo in the stimuli classified as Mahutu is 6.6% slower than in the stimuli identified as Losto, and this difference is below the just noticeable threshold.

Listeners' sensitivity to speech tempo can be explained by a number of studies in physiology of hearing. Schreiner and Urbas (1986, 1988) showed that auditory neurons fire in response to a sharp increase in intensity that usually coincides with the vowel onset. Consequently, the rate at which “*s”* and “*a”* alternate in the stimuli determines the rate at which the neurons fire. Moreover, some studies suggest a direct relation between a syllable-length unit (“*sa”* unit in our stimuli) and the neural response in the auditory cortex (Viemeister, 1988; Greenberg, 1997; Wong and Schreiner, 2003; Greenberg and Ainsworth, 2004). Besides, the auditory system imposes certain limitations on the speech tempo. If the assumptions to the speech rate and to the length of the syllable-like units are violated, speech processing and decoding of speech at the cortical level is compromised (Ghitza and Greenberg, 2009; Ghitza, 2011). Therefore, there is a physiological basis for discriminating fast and slow stimuli, or stimuli with longer and shorter syllable-like units.

We are not aware of any evidence of direct physiological correlates for the ability to differentiate fine distinctions in durational variability. Thus, we assume that differentiation of fine distinctions in rhythmic patterns involves cognitive processing. Peculiarities of predominant rhythmic patterns in a certain language correlate with grammatical, morphological and other structural characteristics. Rhythmic patterns guide the way the language is acquired. They influence the strategies of segmentation of continuous speech. Rhythmic cues are exploited differently by listeners with different native languages for purposes of speech processing (Christophe et al., 2003; Murty et al., 2007; Thiessen and Saffran, 2007; Kim et al., 2008). However, their importance in processing non-linguistic stimuli when cognitive mechanisms are less intensely employed might be low. When the presented stimuli are not processed as speech-like, rhythmic differences between stimuli are not used for discrimination or classification (Ramus et al., 2000). Thus, in our experiment listeners rely more on those patterns in acoustic signal that have direct physiological correlates rather than on the patterns that are processed though the relay of cognitive filters.

We would like to emphasize that our results do not indicate the inability of the participants to hear the rhythmic differences. To test the ability to detect the differences in L2 speech rhythm between proficiency levels, discrimination test is to be carried out, and a number of studies showed that such small differences are detected. Using classification task, we can determine which timing patterns are used to classify the utterances into groups. It is possible, that larger differences in durational variability (e.g., between rhythmically contrastive languages that are traditionally defined as stress-timed and syllable-timed) might become more linguistically relevant and used in classification. Smaller differences as those revealed between L2 varieties are not sufficiently different to be processed as linguistically relevant, and timing patterns are classified based on direct physiological correlates. Further research is necessary to address which rhythmic differences could be processed as linguistically relevant.

## Conclusion

We have shown significant differences in speech timing between the sentences produced by the German learners of English at different proficiency levels. As L2 acquisition progresses, L2 English is delivered at a faster rate and with a higher degree of stress-timing. Further analysis revealed that realization of timing is more stable in L2 speech produced by advanced L2 learners. Advanced learners tend to speak consistently with a higher degree of stress-timing. Lower proficiency speakers randomly vary the degree of durational variability in their speech, sometimes delivering L2 speech with high durational variability, and sometimes with a more syllable-timed rhythm. We suggest that timing control in L2 speech production improves as acquisition progresses, and rhythm becomes more stable.

Although rhythmic changes in L2 acquisition can be easily profiled with normalized rhythm metrics, raw metrics do not exhibit a clear uni-directional development. Faster speech rate at higher proficiency levels lowers the values of raw metrics, while the need to enhance durational variability pushes the metrics up. These conflicting forces did not allow raw metrics to reveal a clear developmental change as a function of acquisition progress.

**Figure 8:**
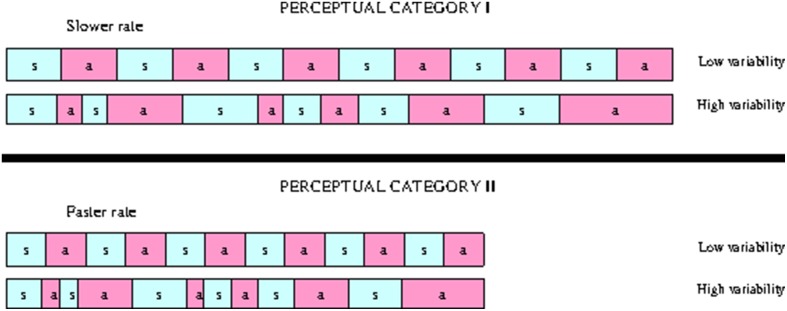
**Splitting the “sasasa” stimuli into two categories based on durational variability of vocalic and consonantal intervals and speech rate**.

Perception experiment was set up to investigate whether monolingual English use the differences in L2 speech timing between proficiency levels to group the utterances with different timing patterns into the same class. Although L2 speech indeed becomes increasingly more stress-timed with proficiency, native speakers of English, when asked to classify different timing patterns into separate groups, paid attention to the differences in speech rate and ignored the differences in speech rhythm between the utterances produced by the L2 learners at different proficiency levels. Faster utterances were grouped separately from slower utterances. Both groups included utterances with high and low durational variability of speech intervals. This trend is schematically illustrated on Figure 8. The sensitivity of the listeners to speech tempo is physiologically determined. The fact that listeners ignore rhythmic differences in classification can be explained by non-linguistic nature of the stimuli. Processing of “sasasa” stimuli in our experiment, assumingly, does not involve cognitive mechanisms that are employed in processing of linguistic material, and listeners pay attention to those features of the acoustic signal that have direct physiological correlates. Further research is necessary to understand whether the cognitive filter is not applied to processing these stimuli because they are not perceived as speech, or because the differences in rhythm between the stimuli are not sufficiently large to be linguistically relevant.

### Conflict of interest statement

The authors declare that the research was conducted in the absence of any commercial or financial relationships that could be construed as a potential conflict of interest.

## Footnotes

^1^According to the evaluators' opinion, we did not have true beginners, and our participants better correspond to the lower-intermediate (B1.1 according to the Common European Framework for Languages), upper-intermediate (B2.1) and advanced (C1.1 and C1.2) levels. CEFL specifies skills the learner should achieve at each of the six levels: A1, A2, B1, B2, C1, C2. However, as each level is usually covered in language schools during two intensive courses, teachers split each level into two sublevels, e.g., C1.1 and C1.2. However, official CEFL guidelines do not split six levels into sublevels, and division into C1.1 and C1.2 is done—rather arbitrarily—by the teachers. We did not want to resort to commonly used placement tests to evaluate the learners' proficiency level because standard placement tests are designed to make an ***initial*** assessment to place the student into the course that fits his level. Placement tests are not designed to estimate the proficiency level for certification of the achieved proficiency level. Therefore, using several human evaluators (to avoid human bias) is the best methodological option, in our opinion.
