# Supplementary material for: Perception of speech rhythm in second language: the case of rhythmically similar L1 and L2
Source: Front Psychol. 2015 Mar 25;6:316. doi: 10.3389/fpsyg.2015.00316 (PMC4373255; doi:10.3389/fpsyg.2015.00316)
Supplement: Supplementary file 1 [file DataSheet1.DOC]

***Appendix I***

***This form is intended to collect information from participants to assess different factors which might affect foreign accent in L2 speakers and learners.***

*Language background questionnaire*

*Contact information*

Name:

e-mail:

***Background information:***

1. Where were you born (please state country and region)?
2. Where did you grow up? Please state county/countries and region/regions where you have lived longer than a year, and write the number of years you spent in each country/region
3. How would you call the variety of German you are speaking (e.g. standard, northern, Bavarian, Austrian, Swiss, etc)?

***Language use*** (tick what is appropriate)

|  | **Only German** | **German and English** | **Only English** | **Other, namely (please specify)** |
| --- | --- | --- | --- | --- |
| **Which language/languages do you use more at home when talking to your mother?** |  |  |  |  |
| **Which language do you use more at home when talking to your father?** |  |  |  |  |
| **Which language do *your parents* use at home?** |  |  |  |  |
| **Which language do you use more at home with your partner?** |  |  |  |  |
| **Which language do you use more at home with your children?** |  |  |  |  |
| **Which language do *YOU* mostly use at home?** |  |  |  |  |

1. Estimate your abilities in English and in other most commonly used languages (on scale from 1=very poor to 7=native or native like)

|  | Language used | | | |
| --- | --- | --- | --- | --- |
| | **Language skills** | | --- | | Speaking | | Understand | | Reading | | Writing | | | **English** | | | | | | | | --- | --- | --- | --- | --- | --- | --- | | 1 | 2 | 3 | 4 | 5 | 6 | 7 | |  |  |  |  |  |  |  | |  |  |  |  |  |  |  | |  |  |  |  |  |  |  | |  |  |  |  |  |  |  | | |  | | | | | | | | --- | --- | --- | --- | --- | --- | --- | | 1 | 2 | 3 | 4 | 5 | 6 | 7 | |  |  |  |  |  |  |  | |  |  |  |  |  |  |  | |  |  |  |  |  |  |  | |  |  |  |  |  |  |  | | |  | | | | | | | | --- | --- | --- | --- | --- | --- | --- | | 1 | 2 | 3 | 4 | 5 | 6 | 7 | |  |  |  |  |  |  |  | |  |  |  |  |  |  |  | |  |  |  |  |  |  |  | |  |  |  |  |  |  |  | | |  | | | | | | | | --- | --- | --- | --- | --- | --- | --- | | 1 | 2 | 3 | 4 | 5 | 6 | 7 | |  |  |  |  |  |  |  | |  |  |  |  |  |  |  | |  |  |  |  |  |  |  | |  |  |  |  |  |  |  | |

1. Have you attempted any standardized language test (e.g. IELTS)? Is so, what test have you attempted and what results have you obtained?
2. Could you please indicate whether you use English or other languages besides German with these people? If something is not applicable to you, please leave the line empty.

|  | Always | Often | Sometimes | Rarely | Never |
| --- | --- | --- | --- | --- | --- |
| With my partner, or last partner |  |  |  |  |  |
| With my children |  |  |  |  |  |
| With my relatives |  |  |  |  |  |
| With my partner’s relatives, or last partner’s relatives |  |  |  |  |  |
| With friends in Germany |  |  |  |  |  |
| With friends abroad |  |  |  |  |  |
| With my co-workers in Germany |  |  |  |  |  |
| With my co-workers abroad |  |  |  |  |  |

1. How did you learn English up to this point (circle what is true for you):
   1. mainly through formal classroom instruction
   2. mainly through interacting with people
   3. a mixture of both
   4. other (please specify):
2. Have you received any formal instruction or training in pronunciation of English? Please specify what kind of instruction and for how long?
3. Which foreign languages did you learn at school/university
4. Do you play any musical instrument?
5. What music and how many hours a week do you listen to?
6. Do you have any musical training (in singing, musical performance?)

***Motivation***

Please describe your feelings about certain things. Please read each statement below and all possible answers, then circle the point on the scale which most accurately reflects your feelings about the statements:

1. I must pronounce English well in order to have or keep a good job and to be respected by people I work with (colleagues/customers):

**NOT true** in my case

Partly true in my case

**True** in my case

1. My social status is determined by how well I can pronounce English

**NOT true** in my case

Partly true in my case

**TRUE** in my case

1. Improving my pronunciation of English can be important to me because
   1. It will enable me to gain good friends more easily among native English speakers

Definitely **not** my feeling

Partly my feeling

Definitely **is** my feeling

- 1. It will allow me to meet and converse with more and varied native English-speaking people

Definitely **not** my feeling

Partly my feeling

Definitely **is** my feeling

- 1. It will enable me to feel and behave like native English-speakers

Definitely **not** my feeling

Partly my feeling

Definitely **is** my feeling

1. If native English speakers began to thing of me as an English, not as a German

I **would not** like it

I would have **mixed** feelings

I **would like** it very much

1. During the years I have been learning English I have been concerned about pronunciation

**Less** than others

**As much** as others

**More** than others

1. Do you think accent-free speech in English is achievable for you?

**NOT true** in my case

Partly true in my case

**True** in my case

1. Native English speakers will not be able to tell my origin by my speech, but they will be able to tell that I am not a native speaker of English

**Not** true in my case

Partly true in my case

**True** in my case

1. Please write down why accent free English is important / not important to you (you can mention several reasons)

***Part V***

Please give your comments on anything you feel important about language background, language use, and language learning:
