# Supplementary material for: Perception of speech rhythm in second language: the case of rhythmically similar L1 and L2
Source: Front Psychol. 2015 Mar 25;6:316. doi: 10.3389/fpsyg.2015.00316 (PMC4373255; doi:10.3389/fpsyg.2015.00316)
Supplement: Supplementary file 2 [file DataSheet2.DOC]

***Appendix II***

***Pronunciation Test (Tester’s Version)***

**55 points maximum for the perception part of the test.**

**Task No. 1**

**A)**

**Listen and mark the word you hear in each line:** 27 points max, one point for each correct answer

thin thing think

win wing wink

pin ping pink

sin sing sink

ban bang bank

banner banger banker

thigh tie sigh

thick tick sick

theme team seem

path part pass

tenth tent tense

fourth fought force

rebel rabble rubble

rebel rabble rubble

rebel rabble rubble

nets gnats nuts

nets gnats nuts

trek track truck

trek track truck

flesh flash flush

flesh flash flush

don't sleep/slip now

here is some cheap/chip oil

what a nice toy ship/sheep

have you got any beans/bins

that pitch/peach is OK

can I have a lick/leek?

**B)**

**Listen to these people greeting each other and mark if they are neutral and uninterested, or friendly and interested, or excited and pleased to see each other** (6 points maximum, 1 point for each correct answer)

| **Conversation number** | **1** | **2** | **3** | **4** | **5** | **6** |
| --- | --- | --- | --- | --- | --- | --- |
| **Neutral and uninterested to see each other** |  |  |  |  |  |  |
| **Friendly and interested to see each other** |  |  |  |  |  |  |
| **Excited and pleased to see each other** |  |  |  |  |  |  |

**C)**

**Listen to the recording and decide if the second speaker is interested in what the first speaker says** (8 points maximum, 1 point for each correct answer)

|  | Second speaker is interested | Second speaker is not interested |
| --- | --- | --- |
| -Last summer we hitchhikes to Turkey  -Did you? |  |  |
| -Yes, and it only took three days  -Did it? |  |  |
| -We were in Hawaii this time last week-  -Were you? |  |  |
| -Mmm, it was absolutely fantastic  -Was it? |  |  |
| -John and Vera had a lovely holiday in Corfu  -Did they? |  |  |
| -Yes, and John took many photographs  -Did he? |  |  |
| -We spent our holiday in Britain this year  -Did you? |  |  |
| -Yes, but it was more expensive than going abroad  -Was it? |  |  |

**D)**

**Listen to the second speaker in the conversations. He always says “OK”. Does he always want what the first speaker suggests? Mark if the second speaker likes or dislikes the idea** (6 points maximum, 1 point for each correct answer)

| **Talk No.** | **1** | **2** | **3** | **4** | **5** | **6** |
| --- | --- | --- | --- | --- | --- | --- |
| **Second speaker likes the idea** |  |  |  |  |  |  |
| **Second speaker dislikes the idea** |  |  |  |  |  |  |

**E)**

**Listen to the sentences and decide if the second speaker sounds friendly or unfriendly** (8 points maximum, 1 point for each correct answer)

|  |  | Polite and friendly | Not very friendly |
| --- | --- | --- | --- |
| 1 | -Mr. Smith?  -Good morning. Do come in. |  |  |
| 2 | -Aylesbuty Electricals. Can I help you?  -I'd like to speak to Martin Turner, if he's available |  |  |
| 3 | -Where do you live in England?  -I live in Wales, actually |  |  |
| 4 | -Do you know where the post office is?  -I don't, I am afraid |  |  |
| 5 | -Can I take this chair?  -Sorry, somebody's sitting there, actually |  |  |
| 6 | -Good morning.  -Ah, you are the new secretary, aren't you? |  |  |
| 7 | -Yes?  -Coffee, please |  |  |
| 8 | -Good morning.  -Do sit down |  |  |

**55 points maximum for the perception part of the test.**

**100 points maximum for the production part**

**Read the following sentences:**

**A)** *The rater pays attention only to* *the pronunciation of the prepositions, 2 points for each correctly pronounced contrastive pair, max 10 points*

Where are they from?

They are from Spain

Who did we speak to?

We spoke to Jane

What is it made of?

It is made of plastic

Who is she waiting for?

She is waiting for Frank

Who is he looking at?

He is looking at Andreas.

**B)** *The rater watches only for the pronunciation of the modal verbs, 1 point for each correctly pronounced contrastive pair, max 4 points*

I can speak English but I can't speak German

I can drive a car but I can't drive a lorry

I can read fast but I can't write fast

I can play football but I can't play tennis

**C)** *The rater pays attention only to* *pronunciation of the prepositions, 2 points for each correctly pronounced preposition, max 12 points*

I would like a cup of coffee and after that a glass of wine. A piece of cake with coffee is nice, too. And a box of chocolate, please.

I'm going out to buy a newspaper.

I am going to the airport to meet some friends

**D)** *Watch for pronunciation of the linkings, elisions and loss of plosions (in bold), 1 points for each correctly pronounced phenomenon, max 16 points*

He likes tenni**s a**n**d** skiing

My favouri**te** colour**s a**re yello**w a**n**d** green

He's very friendl**y a**n**d** talkative

She tol**d** me to carr**y e**verything

Ho**w a**re you?

He promise**d** to men**d** my bike

She le**t** them rea**d** fo**r a** while

**E)** *Watch for loss of plosions (in bold), 1 point for each loss, max. 6 points*

Rose is our younge**st d**aughter

She is wearing a re**d T**-shirt

It's a bi**g co**mputer company

His alarm cloc**k g**oes off at eight

I love ri**pe b**ananas!

What are my jo**b p**rospects?

**F)** *Watch for pronunciation of the modal verbs, 1 point for each correctly pronounced modal verb and 1 point for each correctly pronounced stress, max 12 points*

*TASK FOR THE PARTICIPANT:*

**Read these sentences and make the words in bold stressed**

I must **email** you

You should tell **him** about it

I have to go home **now**

He has to work **very** hard

I had to get up at **six**

I must be **there** at three

**G)** *Watch for assimilations, 2 points for each correctly pronounced cluster, 14 points max.*

Make sure everything is i**n p**lace, i**n c**ase they arrive early.

I spent half the year i**n P**aris and the rest i**n B**erlin

Careful on that street. There is a lot of ba**d g**uys there.

The Pri**me m**inister is due to visit Russia.

There were si**x st**udents waiting for the teacher

**H)** *General assessment of pronunciation skills (from 1 to 6 points maximum for all the sentences)*

-I am sure we're being followed, you know... you see that black car – it's been driving behind us for ages.

-Don't be ridiculous – why would anyone want to follow us? You're just being paranoid.

-What a mess!!! What on earth have you been doing?

-We were only trying to make a nice surprise for you.

-Excuse me, I was told I'd be seen immediately, and I've been waiting for ages now!

-The doctors are doing their best, but I'm afraid they're very busy. Do you think you could wait over there, please?

**I)**

**Read these sentences first politely and the in a rude manner**

*(5 points maximum, 0.5 points for each production)*

Would you mind waiting a moment?

Do you think you could possible help me?

Can you bring the manager here, please?

Would you mind keeping the noise down?

Could you look after Rose a minute?

**G)**

**Read these short conversations.**

*6 points maximum, 1 point for each pair of contrastive sentences, the rater should evaluate contrastive stress.*

A: So, you've been collecting coins for 10 years...

B: Well, no, actually, I've been collecting stamps for 10 years.

A: So you've been smoking since you were 16...

B: Well, no, actually, I've been smoking since I was 11.

A: So, you been driving for 6 years...

B: Well, no, actually, I've been driving for 6 months.

A: so you've been drinking my beer by mistake...

B: Well, no, actually, Pete's been drinking your beer by mistake.

A: So, you've been writing poems for 20 years

B: Well,no, actually, I've been writing poems fro 30 years.

A: So, you've been relaxing in Prague since April...

B: Well, no, actually, I've been working in Prague since April.

**K)**

**Read the chunks in italic to express the meaning in the brackets**

*9 points maximum, 1 point for each chuck, the rater pays attention to intonational patterns*

Teenagers! I never know when Mark's going to get home.

*Tell me about it* (You don't have to tell me, I know)

When I win a lottery, I'll buy a luxury boat

*You wish!* (You don't have a chance)

Would you like to go out with me?

*In your dreams* (it is never going to happen)

Do we have a chance of winning?

*No way!* (Definitely not)

What are you going to study when you get to the university?

*As if!* (That is unlikely)

What will you do if you won a lottery?

*I should be so lucky* (That would be great but it will never happen)

I am sure the next President will be a woman

*Yeah, right* (not a chance)

Well, think we should all stay late

*Speak for yourself* (That is your opinion, but I disagree)

**100 points maximum for the production part**
