# Supplementary material for: Perception of speech rhythm in second language: the case of rhythmically similar L1 and L2
Source: Front Psychol. 2015 Mar 25;6:316. doi: 10.3389/fpsyg.2015.00316 (PMC4373255; doi:10.3389/fpsyg.2015.00316)
Supplement: Supplementary file 3 [file DataSheet3.DOC]

***Appendix III***

| **Task and task number** | **First attempt** | **Second attempt** | **Neither** | **NAME**  **and comments** |
| --- | --- | --- | --- | --- |
| **1) alveolar affricate production**  **atso** |  |  |  |  |
| **2) Bilabial frivative voiceless production**  **aфo** |  |  |  |
| **3) voiceless nasal production**  **ammo** |  |  |  |
| **4) long bilabial plosive production**  **appo** |  |  |  |
| **5) bilabial click production**  **a#o** |  |  |  |
| **6) high-low-high lexical tone production**  **mo (tone on the vowel)** |  |  |  |
| **7) voiceless lateral production**  **allo** |  |  |  |
| **8) ingressive airstream production**  **'hello' on ingressive airstream** |  |  |  |
| **9) falling tone**  **Say 'this way' with a falling tone (when giving instruction produce 'this way' with rising tone)** |  |  |  |
| **10) rising tone**  **Say 'that way' with a rising tone (when giving instruction produce 'this way' with falling tone)** |  |  |  |
| **11a) clap your hands and repeat the rhythm**  **-...--** |  |  |  |
| **11b) clap your hands and repeat the rhythm**  **...--..** |  |  |  |
| **11c) clap your hands and repeat the rhythm**  **--...-** |  |  |  |
| **12a) use clicks to produce these patterns**  **..--..** |  |  |  |
| **12b) use clicks to produce these patterns**  **-..--...** |  |  |  |

The participant imitates the sounds which are produced by the phonetician. If the participant is successful, he receives two points and the rater ticks the mark in the first column. If the participant is not successful, the phonetician produces the sound once again, and the participant attempts one more time. If his second attempt is successful, the phonetician ticks the mark in the second column, and the participant receives one point. Otherwise the phonetician ticks the mark in the third column and moves on to the next sound.

The participant can score maximum 30 points in this test.
