# Supplementary material for: Perception of speech rhythm in second language: the case of rhythmically similar L1 and L2
Source: Front Psychol. 2015 Mar 25;6:316. doi: 10.3389/fpsyg.2015.00316 (PMC4373255; doi:10.3389/fpsyg.2015.00316)
Supplement: Supplementary file 4 [file DataSheet4.DOC]

***Appendix IV***

***Questions for the Interview***

1. Where do you come from? Tell me about this place.
2. Do you speak other languages besides English and German? How often? How did you learn these languages?
3. Have you ever lived in the UK or the USA? Tell me about your experience. Where would you like to go? Why?
4. Which language do you speak at home?
5. Do you play a musical instrument? How often? When did you start playing?
6. Which music do you like? How often do you listen to music?
7. What do you study? Why did you choose this discipline?
8. Is pronunciation of English important to you? Why?
9. How do you learn English? What do you do to brush it it?
