# Supplementary material for: Perception of speech rhythm in second language: the case of rhythmically similar L1 and L2
Source: Front Psychol. 2015 Mar 25;6:316. doi: 10.3389/fpsyg.2015.00316 (PMC4373255; doi:10.3389/fpsyg.2015.00316)
Supplement: Supplementary file 5 [file DataSheet5.DOC]

***Appendix V***

Elicited sentences for analysis

1. the dog is eating a bone *
2. the book is on the table *
3. the girl is eating an apple *
4. the ball is on the chair *
5. the boy is kicking the ball *
6. the man is catching a fish
7. the knife is on the table *
8. the children are watching the giraffe
9. the boy is drinking juice
10. the bread is on the table *
11. the boy is eating ice-cream
12. the boy is under the tree
13. the cat is drinking milk *
14. the girl is talking on the phone
15. the girl is buying a balloon
16. the woman is eating an orange
17. the baby is crying *
18. the baby is sleeping *
19. the cat is chasing the mouse *
20. the man is walking *
21. the dog is chasing the car
22. the baby is taking a bath
23. the boy is riding a bike
24. it's raining outside *
25. the girl is eating candy
26. the children are going to school
27. it's snowing outside *
28. the dog is chasing the cat
29. the spoon is on the table *
30. the boy is running to the car
31. the boy is talking on the phone
32. the girl is chasing the boy
33. the computer is on the table

The sentences marked with asterisk were chosen to convert into the stimuli for the perception experiment.

Example of a picture used for sentence elicitation task. 26 pictures were used from the study of Bunta and Ingram (2007). This set was extended by 7 more pictures drawn in similar manner and style (for sentences 27-33) to reach the desired number of 33 sentences.


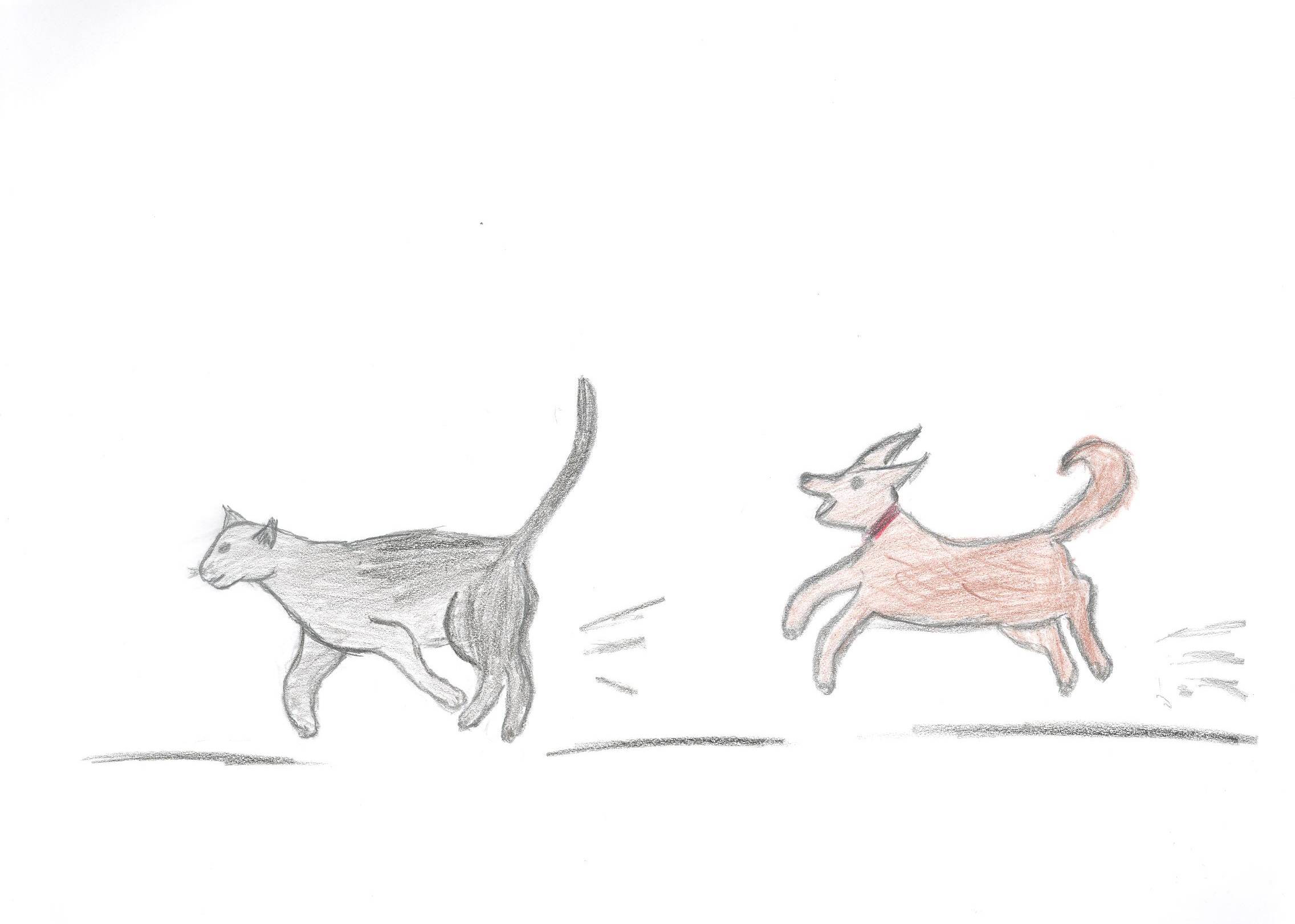


The dog is chasing the cat


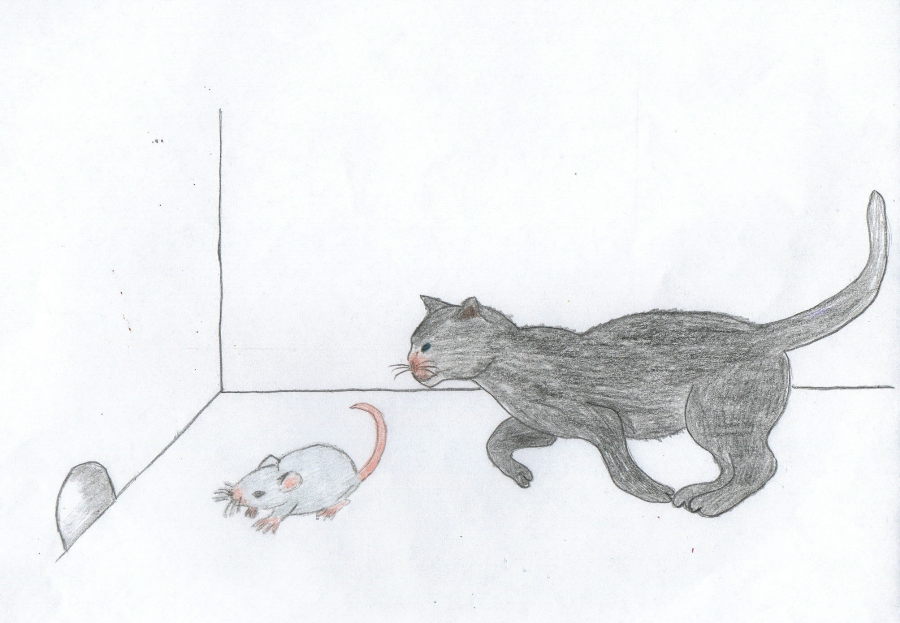


The cat is chasing the mouse
